# Supplementary material for: Pro-Apoptotic Potential of Pseudevernia furfuracea (L.) Zopf Extract and Isolated Physodic Acid in Acute Lymphoblastic Leukemia Model In Vitro
Source: Pharmaceutics. 2021 Dec 16;13(12):2173. doi: 10.3390/pharmaceutics13122173 (PMC8703293; doi:10.3390/pharmaceutics13122173)
Supplement: Supplementary file 1 [file pharmaceutics-13-02173-s001.zip › pharmaceutics-1480994-supplementary.pdf]

pharmaceutics

MDPI

Supplementary Materials: Pro-Apoptotic Potential of *Pseudevernia furfuracea (L.) Zopf* Extract and Isolated Physodic Acid in Acute Lymphoblastic Leukemia Model In Vitro

Martin Kello, Tomas Kuruc, Klaudia Petrova, Michal Goga, Zuzana Michalova, Matus Coma, Dajana Rucova and Jan Mojzis

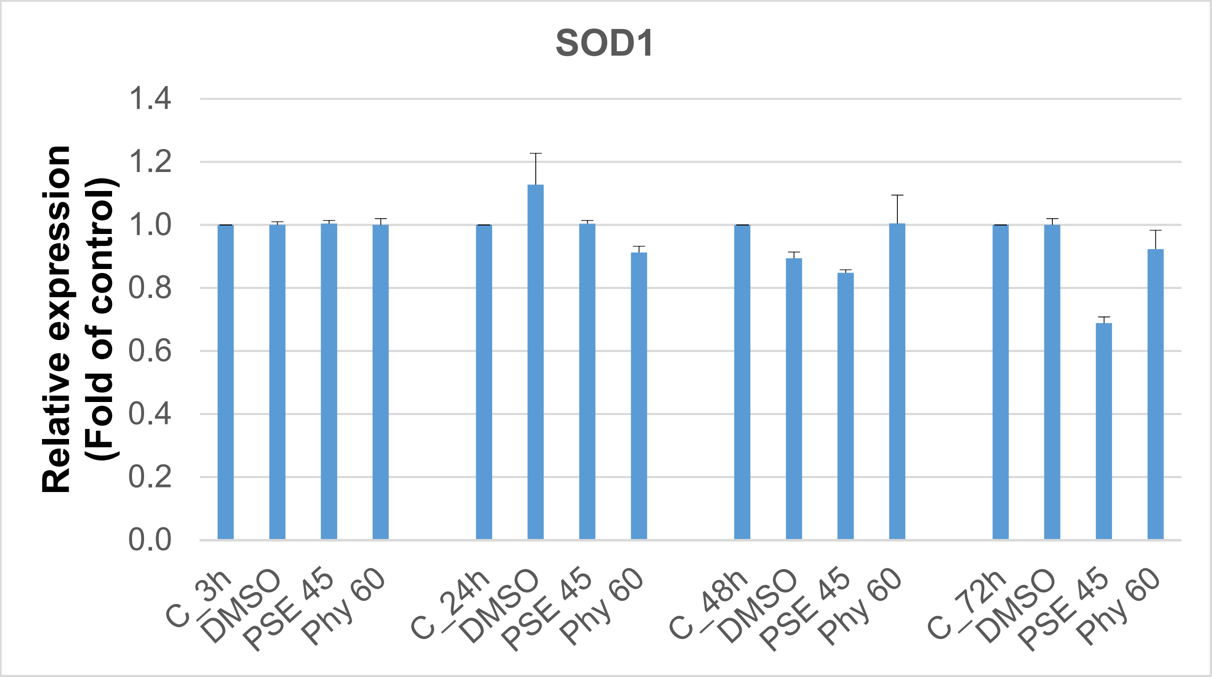

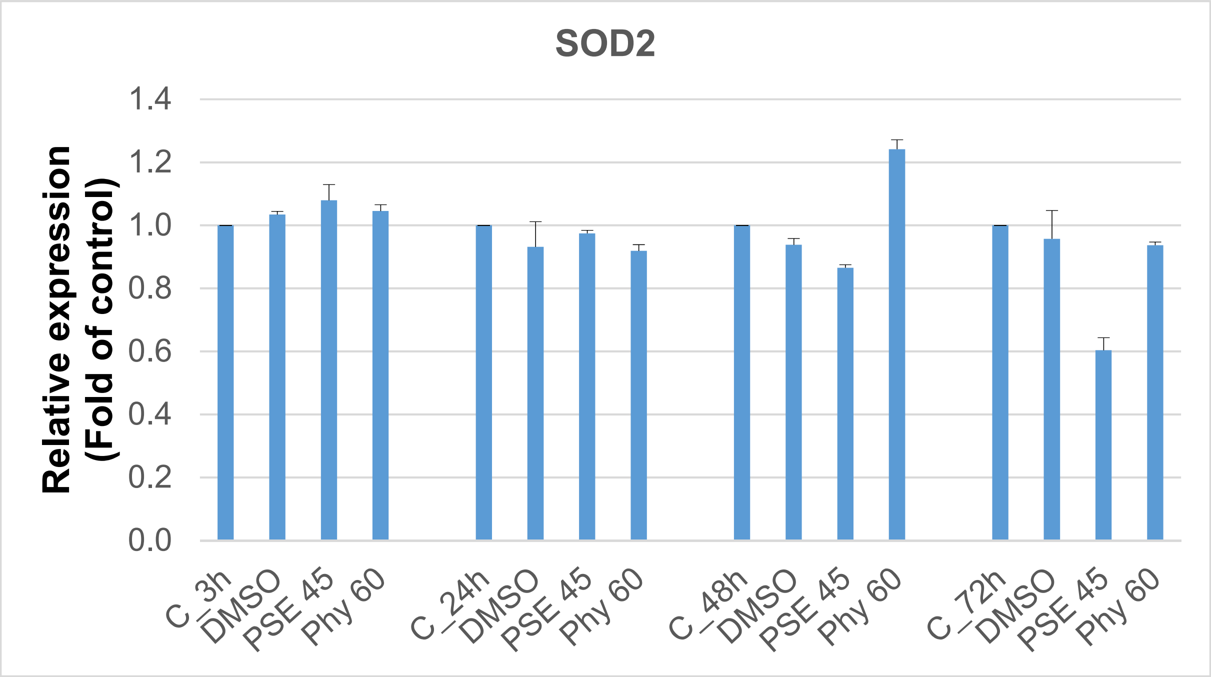

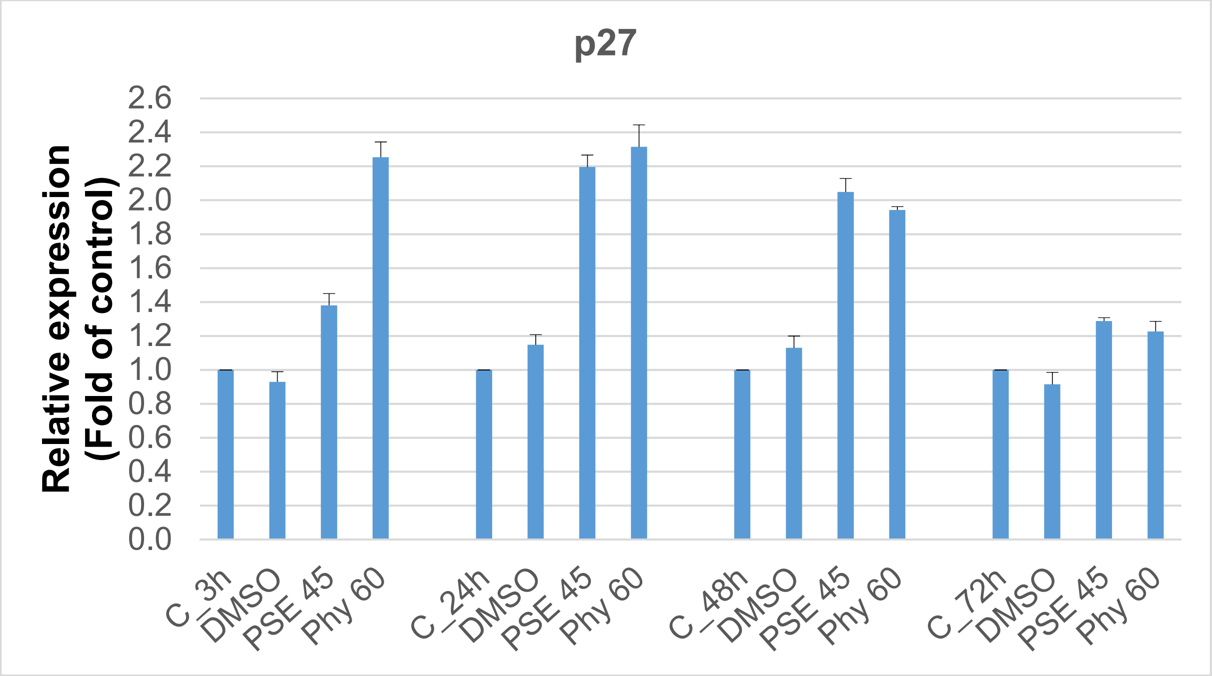
p27
Relative expression
(Fold of control)
2.6
2.4
2.2
2.0
1.8
1.6
1.4
1.2
1.0
0.8
0.6
0.4
0.2
0.0
C_3h
DMSO
PSE 45
Phy 60
C_24h
DMSO
PSE 45
Phy 60
C_48h
DMSO
PSE 45
Phy 60
C_72h
DMSO
PSE 45
Phy 60

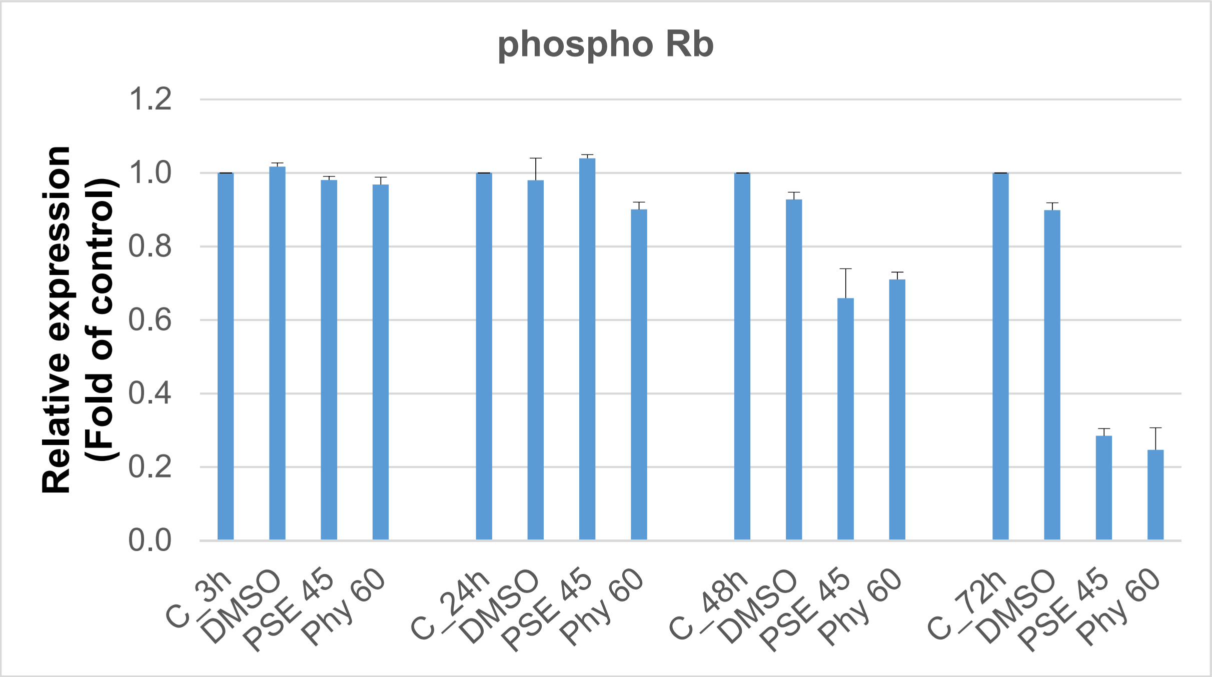
phospho Rb
Relative expression
(Fold of control)
1.2
1.0
0.8
0.6
0.4
0.2
0.0
C_3h
DMSO
PSE 45
Phy 60
C_24h
DMSO
PSE 45
Phy 60
C_48h
DMSO
PSE 45
Phy 60
C_72h
DMSO
PSE 45
Phy 60

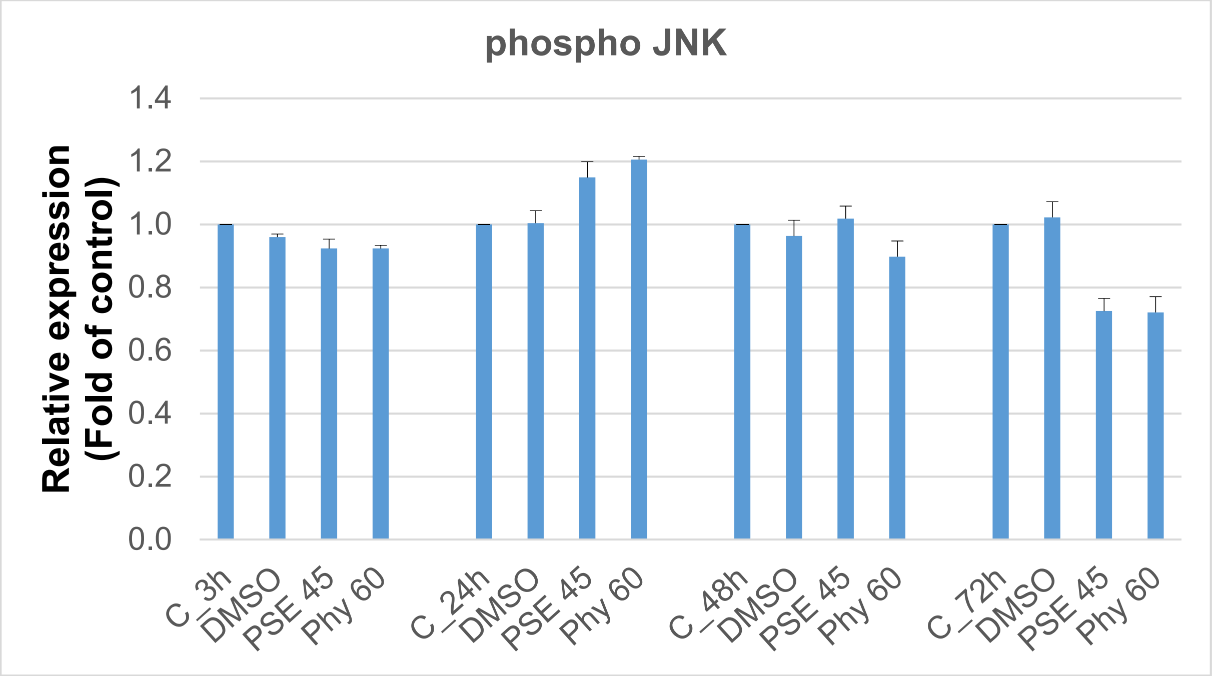
phospho JNK
Relative expression (Fold of control)
1.4
1.2
1.0
0.8
0.6
0.4
0.2
0.0
C_3h
DMSO
PSE 45
Phy 60
C_24h
DMSO
PSE 45
Phy 60
C_48h
DMSO
PSE 45
Phy 60
C_72h
DMSO
PSE 45
Phy 60

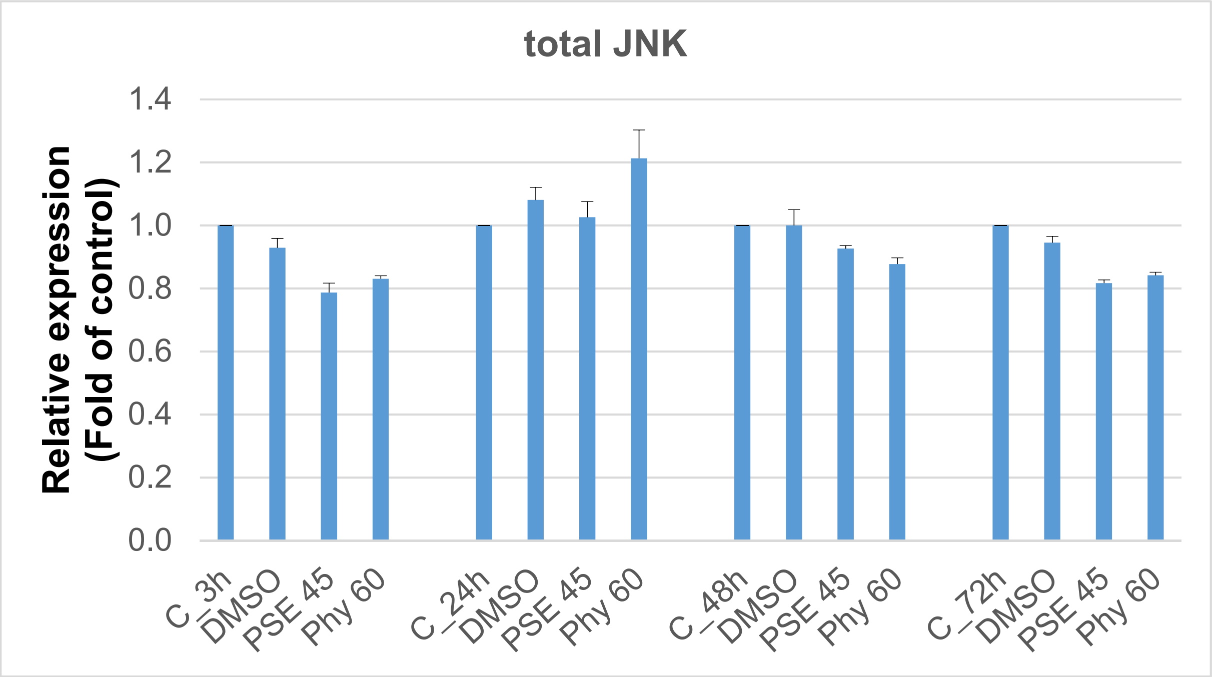
total JNK
Relative expression (Fold of control)
1.4
1.2
1.0
0.8
0.6
0.4
0.2
0.0
C_3h
DMSO
PSE 45
Phy 60
C_24h
DMSO
PSE 45
Phy 60
C_48h
DMSO
PSE 45
Phy 60
C_72h
DMSO
PSE 45
Phy 60

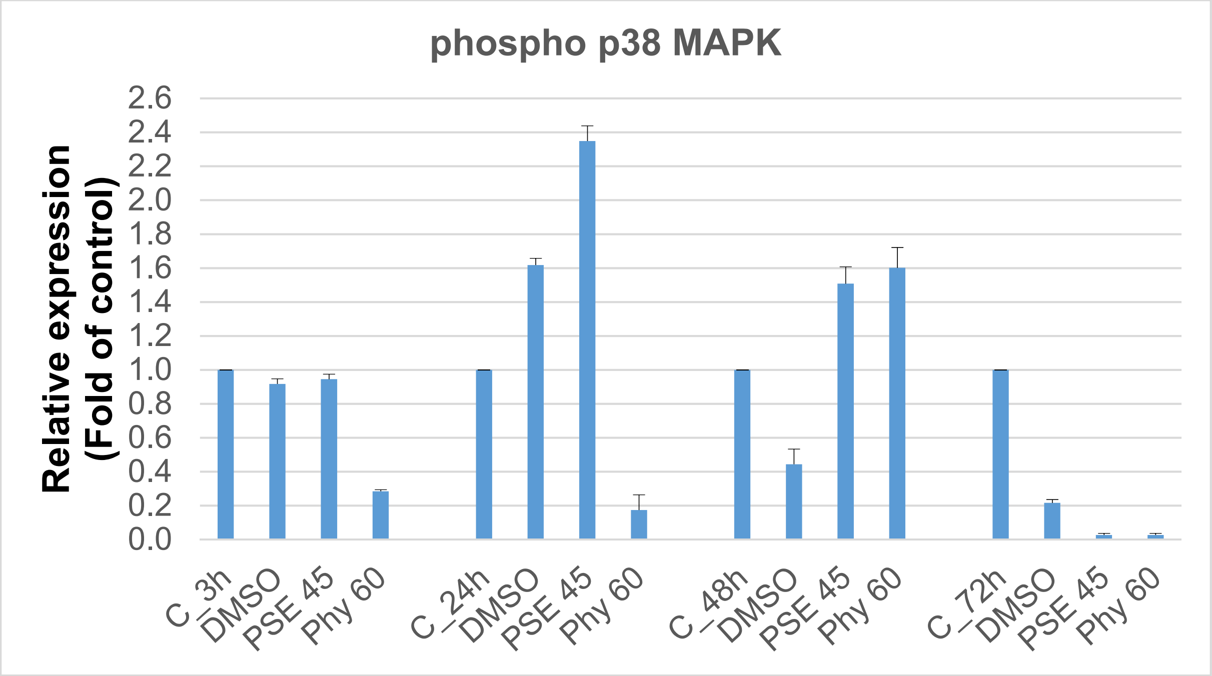
phospho p38 MAPK
Relative expression
(Fold of control)
2.6
2.4
2.2
2.0
1.8
1.6
1.4
1.2
1.0
0.8
0.6
0.4
0.2
0.0
C_3h
DMSO
PSE 45
Phy 60
C_24h
DMSO
PSE 45
Phy 60
C_48h
DMSO
PSE 45
Phy 60
C_72h
DMSO
PSE 45
Phy 60

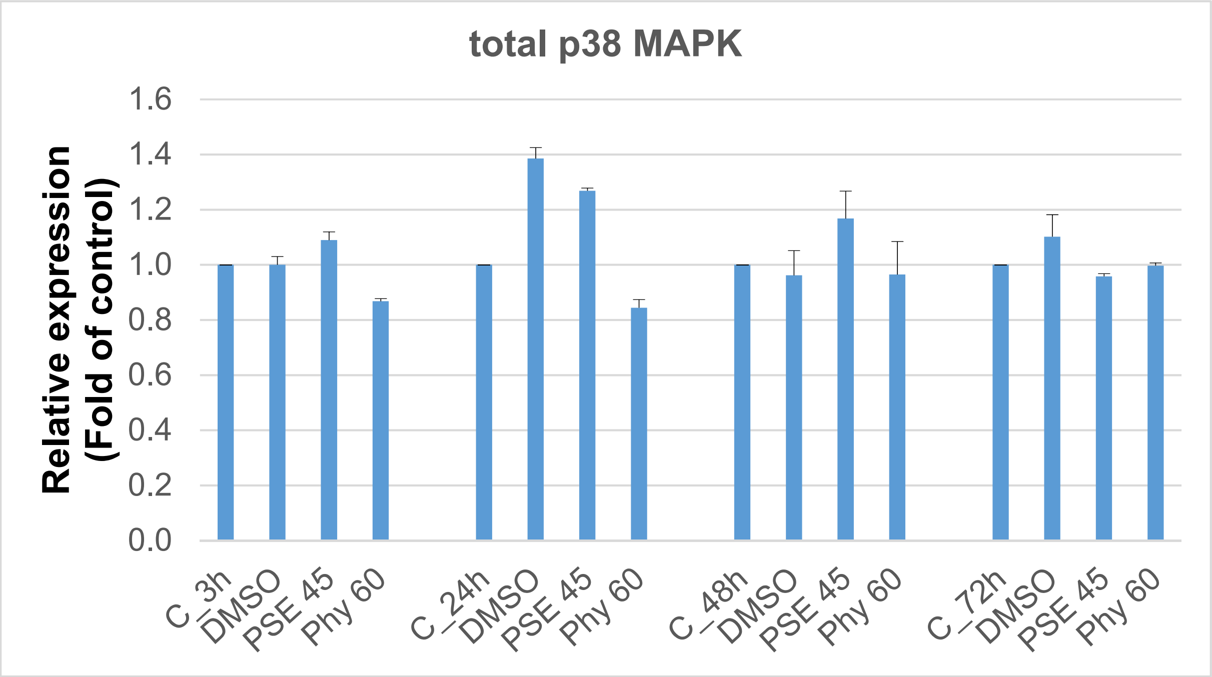
total p38 MAPK
Relative expression
(Fold of control)
1.6
1.4
1.2
1.0
0.8
0.6
0.4
0.2
0.0
C_3h
DMSO
PSE 45
Phy 60
C_24h
DMSO
PSE 45
Phy 60
C_48h
DMSO
PSE 45
Phy 60
C_72h
DMSO
PSE 45
Phy 60

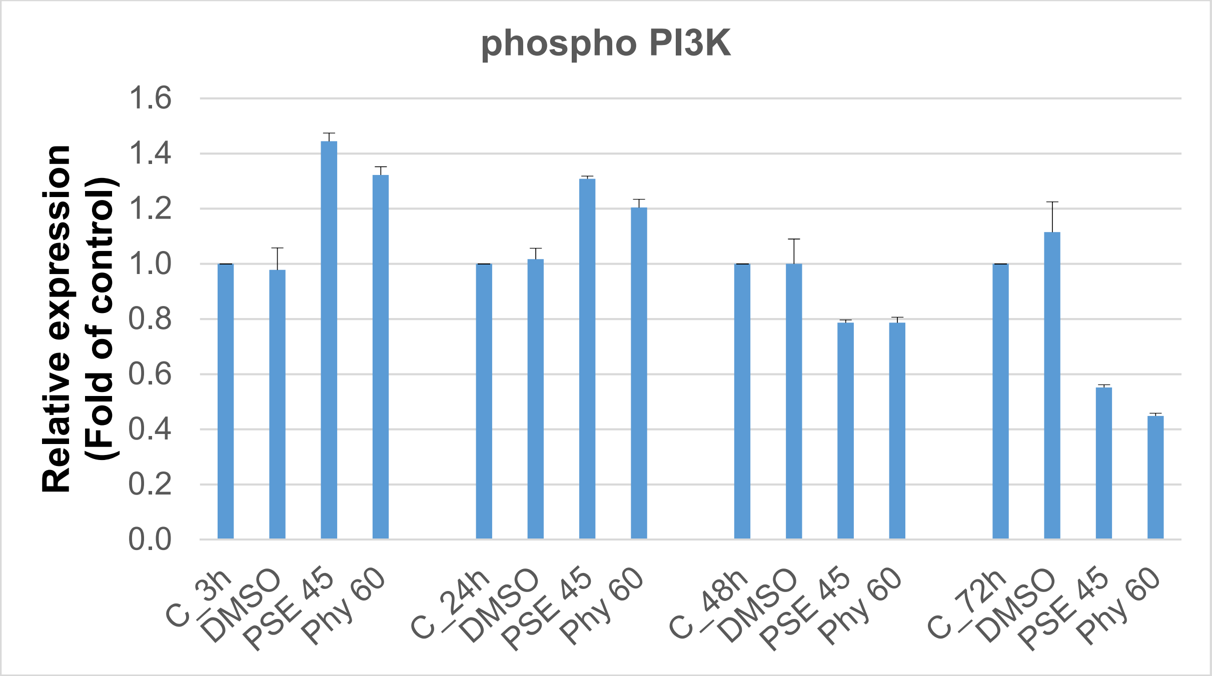
phospho PI3K
Relative expression
(Fold of control)
1.6
1.4
1.2
1.0
0.8
0.6
0.4
0.2
0.0
C_3h
DMSO
PSE 45
Phy 60
C_24h
DMSO
PSE 45
Phy 60
C_48h
DMSO
PSE 45
Phy 60
C_72h
DMSO
PSE 45
Phy 60

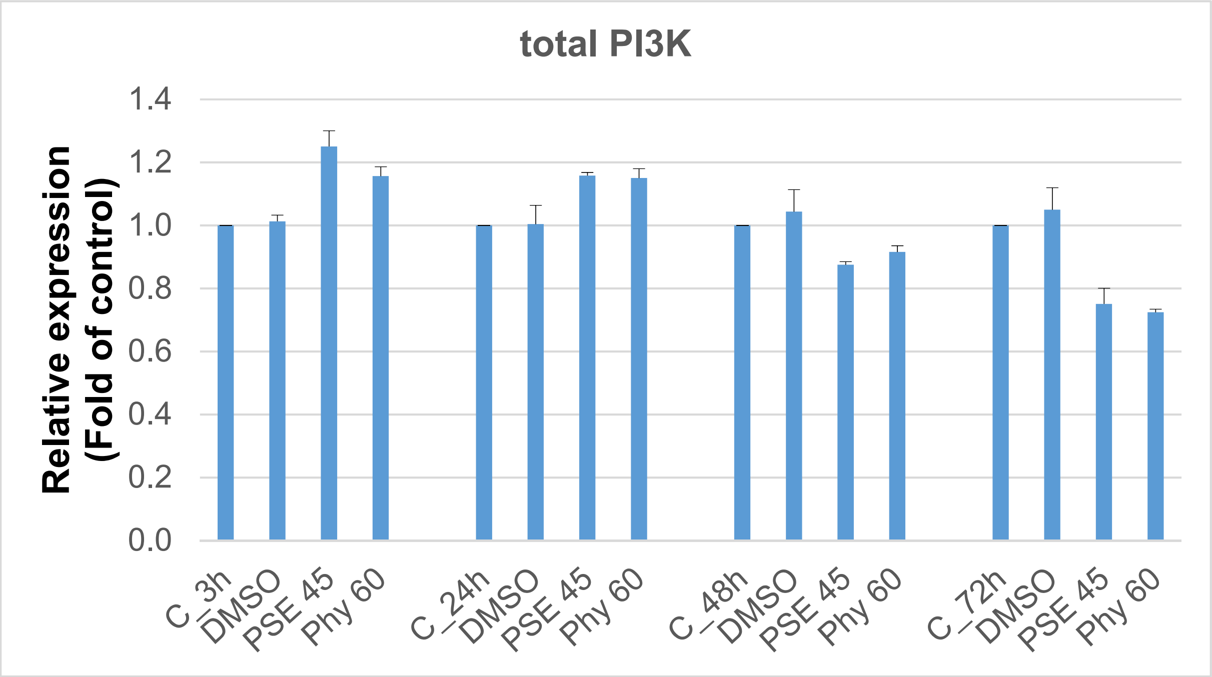
total PI3K
Relative expression
(Fold of control)
1.4
1.2
1.0
0.8
0.6
0.4
0.2
0.0
C_3h
DMSO
PSE 45
Phy 60
C_24h
DMSO
PSE 45
Phy 60
C_48h
DMSO
PSE 45
Phy 60
C_72h
DMSO
PSE 45
Phy 60

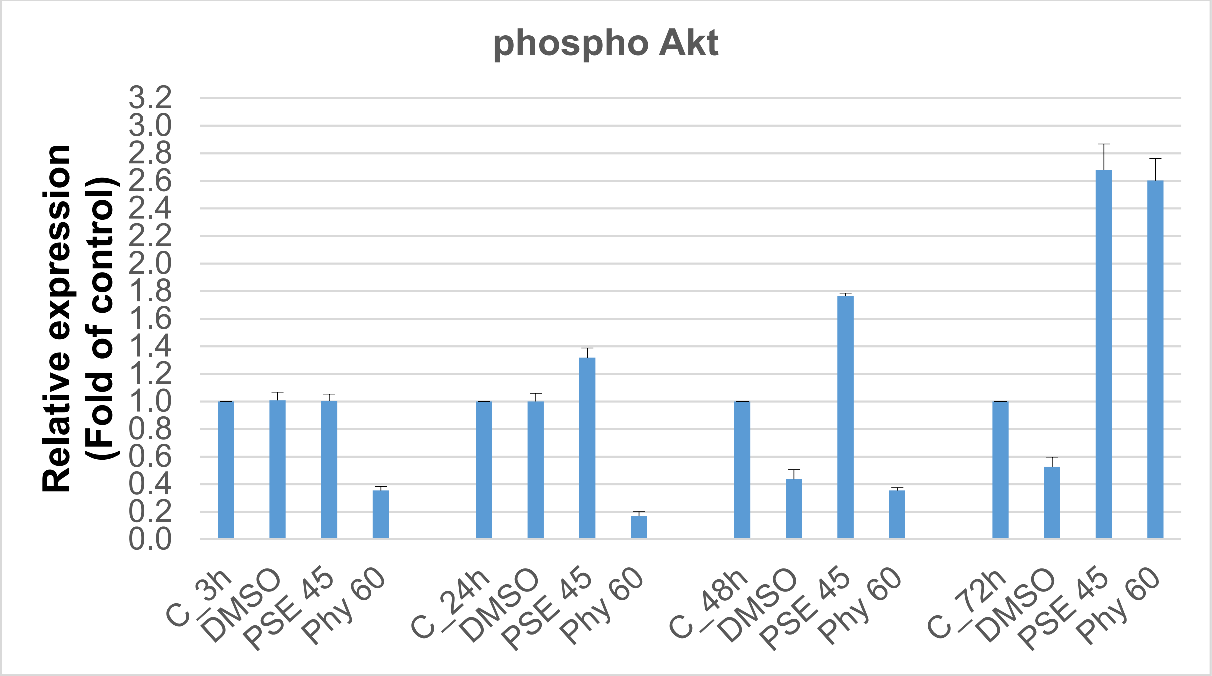

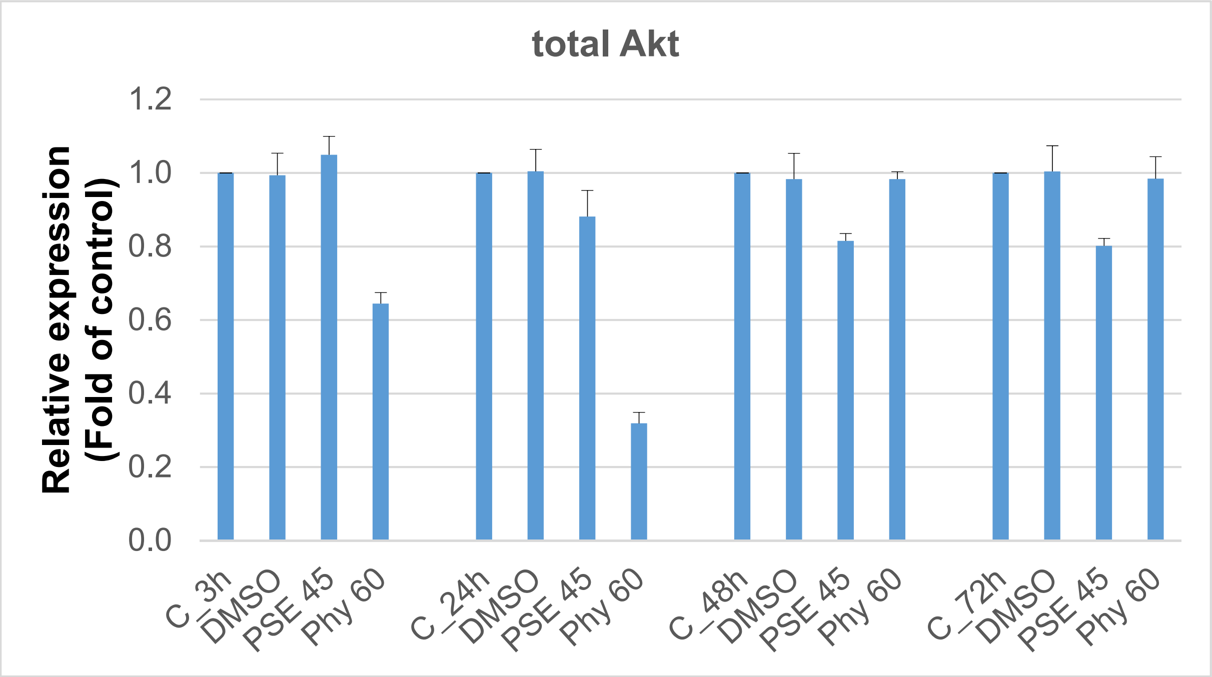

Supplementary file S1：Densitometry graphs from all Western blot analyses.
